# Adrenergic Response to Maximum Exercise of Trained Road Cyclists

**DOI:** 10.2478/hukin-2014-0012

**Published:** 2014-04-09

**Authors:** Grażyna Janikowska, Aleksandra Kochańska - Dziurowicz, Aleksandra Żebrowska, Aleksandra Bijak, Magdalena Kimsa

**Affiliations:** 1 Department of Analytical Chemistry, Medical University of Silesia, Poland.; 2 Chair of Department of Radioisotope Diagnostic and Radiopharmaceuticals, Medical University of Silesia, Poland.; 3 Chair of Department of Physiology, The Jerzy Kukuczka Academy of Physical Education, Poland.; 4 Department of Radioisotope Diagnostic and Radiopharmaceuticals, Medical University of Silesia, Poland.; 5 Department of Molecular Biology, Medical University of Silesia, Poland

**Keywords:** epinephrine, microarray analysis, incremental test

## Abstract

The aim of this study was to evaluate adrenergic responses in the peripheral blood of trained road cyclists at rest, at maximal intensity of incremental bicycle exercise test, and during 15 minutes of recovery, as well as the relationship between them. Competitive male road cyclists, in the pre-competitive phase of a season, mean age 21.7 ± 6.4 years, and BMI 20.7 ± 0.8 kg·m^−2^, performed an incremental test on a bicycle ergometer with unloaded cycling for 5 min, then increased the load to 40 W every 3 min, up to maximal exercise intensity. The plasma catecholamine concentrations (epinephrine, norepinephrine) and oxygen uptake were estimated. The expression of 132 genes related to the adrenergic system in leukocytes was measured. A statistically significant increase in plasma epinephrine concentration (p < 0.01) was observed in response to exercise. The mean of maximal oxygen uptake was 65.7 ± 5.5 ml·kg^−1^·min^−1^. The RGS2 gene expression was highest regardless of the test phase for all athletes. The effort had a statistically significant influence on ADRB2 and RAB2A expression. In addition, the RAB2A, ADM and HSPB1 expression level increased during recovery. We can conclude that plasma epinephrine concentration and genes related to the adrenergic system such as ADM, ADRB2, CCL3, GPRASP1, HSPB1, RAB2A, RGS2 and ROCK1 seem to have an influence on the response to high-intensity exercise in trained cyclists.

## Introduction

The effect of exercise on the organism can be seen in the adrenergic system in the form of increased catecholamine secretion and adrenergic receptor level and other related molecules (Charkoudian and Rabbitts, 2009; Carlson et al., 2011). The activation of a variety of signalling pathways associated with adrenergic responses depends not only on the concentration of agonists (epinephrine or norepinephrine), but also on the amount of receptors and the relationships between them at both the protein and gene levels (Barnes, 1995). The regulation of the sympathoadrenergic system plays an essential role in physiological adaptation to exercise (Brownley et al., 2003). Many of adaptive responses are mediated by increased catecholamine concentrations (Zouhal et al., 2008) and modified adrenergic receptor density and responsiveness (Brownley et al., 2003). Several investigations have suggested that exercise-induced upregulation of the β - adrenergic receptors and the activation of adrenergic system gene expression may be an early adaptive response of sympathetic nerves to endurance exercise (Antezana et al., 1992). Physical training induces changes in the distribution of β adrenergic receptors in several tissues and cell types including the myocardium, adipocytes, and macrophages, which may be important in cardiovascular, metabolic, and immune adaptation to exercise (Schaller et al., 1999; Fuji et al., 1997). Different types of physical activity can change the expression of genes, especially those involved in ventricular function, vasodilator effects, inflammatory response, muscle growth, proliferation, and apoptosis (Carlson et al., 2011). It has been documented that the expression of genes related to the adrenergic system, including β2-adrenoceptor (ADRB2), results in cardiopulmonary adaptation in both untrained and trained subjects. The ADRB2, expressed in the human heart, has been shown to increase left ventricular function and vascular regulation at rest and during exercise. Several studies have indicated than an increase in ADRB2 density in the pulmonary lymphocytes may improve the pulmonary response to prolonged high-intensity exercise (Barnes, 1995; Carlson et al., 2011; Fujii et al., 1997; Schaller et al., 1999; Fragala et al., 2011).

The above-mentioned findings contribute to our understanding of the importance and function of the adrenergic response to exercise that may be involved in physiological adaptation mechanisms. However, there is also evidence that long-term exposure of adrenergic receptors to high levels of catecholamines, generated during exercise, results in down-regulation and desensitization of these receptors, which may be a symptom of overload or an early phase of overtraining (Barnes, 1995; Urhausen et al., 1995). Various types of exercises and their intensity can induce changes in blood variables such as plasma catecholamine concentrations and gene expression. The evaluation of the relationships between the gene that encodes the ADRB2 and genes that encode other proteins might be important for understanding of physiological reactions to physical effort. Thus, the aim of this study was to evaluate the adrenergic responses in the peripheral blood of trained road cyclists before and after maximum physical exercise. This was done through the determination of the relationship between plasma catecholamine concentrations and the expression of 132 genes related to the adrenergic system in leukocytes.

## Material and Methods

### Subjects

Competitive male, road cyclists in the pre-competitive phase of a season (n=14), mean age 21.7 ± 6.4 years, body mass 63.0 ± 1.6 kg, and BMI 20.7 ± 0,8 kg·m^−2^ volunteered to participate in the experiment. All of them had been training for 6.2 ± 2.3 years in the same cycling team. The relative intensity and volume of monthly road training were 1653 ± 110 km. All athletes underwent medical evaluations during the time of the pre-season, which included clinical history and a physical examination. The experimental procedures and possible risks were explained to all participants verbally and in writing, and the participants then gave informed written consent. The experiment was approved by the Ethics Committee of the Medical University of Silesia in Katowice KNW/0022/KB1/122/I/09 and conformed to the standards set by the Declaration of Helsinki.

### Study design

For three days before the experiment, the subjects were on a standardized normocaloric diet with 50–60% carbohydrate, 15–20% protein, and 20–30% fat (37 kcal·kg^−1^) a day. They were also asked to abstain from strenuous exercise. No caffeine, tea, alcohol, medication, juices or other products that could affect gene expression were permitted during the 48 hours before the experiment. On the day of exercise testing, the subjects reported to the Laboratory after an overnight fast. Blood samples were taken in the morning (between 8.00 and 9.00 am) to avoid the effects of diurnal differences in hormone concentrations, from a peripheral catheter inserted into the cubital vein. All the athletes performed a graded cycle ergometer exercise test using an F-Lode Excalibur (USA). The exercise started with unloaded cycling for 5 min, then increased by 40 W every 3 min, up to maximal exercise intensity, while maintaining 60 – 70 rpm. The criteria for maximal intensity and test termination were a respiratory quotient greater than 1.1, a heart rate of 180 beats per minute (bpm), and/or physical exhaustion. All subjects performed the exercise to individual maximal power output (400 ± 40W).

Oxygen uptake (VO_2_) was measured continuously from the 6th minute prior to exercise and throughout each stage of the exercise protocol using the Oxycon Apparatus (Jaeger, Germany).

For the assessment of blood morphological variables, epinephrine and norepinephrine concentrations and gene expression, venous blood samples were collected at rest in a sitting position, 15 minutes before exercise (Ts), at maximal exercise intensity (Te), and after 15 minutes of recovery (Tr).

### Estimation of blood variables and plasma catecholamine concentrations

Morphologic variables in the whole blood of fourteen subjects (n=14) were measured with the ABX MICROS 60 (HORIBA ABX SAS) analyzer, using standard methods and reagents.

Epinephrine (E) and norepinephrine (NE) were extracted from the plasma of each subject, and their concentrations were determined twice using the radioimmunoassay method. The KIPL 0100 and KIPL 02000 kit by BioSource Europe S.A. was used. Radioactivity of the obtained complexes was measured using a Wallac Wizard 2470 automatic gamma counter. The limits for detection in plasma were 0.04 nmol·L^−1^ and 0.22 nmol·L^−1^ for E and NE, respectively. The changes in plasma volume were taken into account in the determination of hormone concentrations after the exercise.

### Gene expression estimation

Immediately after the collection of whole blood, the leukocytes of all subjects were separated. From the obtained leukocytes, the total RNA was isolated according to the manufacturer’s protocol of Invitrogen Life Technologies (USA), using a ready to use reagent mixture called TRIZOL®. The obtained RNA was purified on RNeasy Mini Kit columns from Qiagen (Hilden, Germany). The nine HG-U133A microarrays (Affymetrix Inc., USA) were analysed. The 8 μg of pure RNA that were isolated were used to synthesize double-strand cDNA with a SuperScript Choice System from Invitrogen Life Technologies (California, USA). The synthesis of biotinylated cRNA used the BioArrayHighYield RNA Transcript Labeling Kit from Enzo Life Science (New York, USA), and the fragmentation was carried out using a set of reagents from Sample Cleanup Module Qiagen Gmbh (Germany). A GeneChip® Expression, 3′ - Amplification Reagents Hybridization Control Kit was used for hybridization of the obtained sample with HG-U133A microarray, according to the protocol of Affymetrix Inc. Manual of Technical Gene Expression Analysis (California, USA). Microarrays were read immediately after their execution procedures using the GeneArray Scanner 3000 7G (Affymetrix Inc.). Subsequent steps of the preparation of RNA, cDNA and cRNA were monitored qualitatively by the agarose (1%) electrophoresis technique and their quantities by using a GeneQuant II spectrophotometer-calculator (Pharmacia LKB Biochrom Ltd, Cambridge, UK). A preliminary analysis of the results was carried out in the Affymetrix Data Mining Tool and MicroArray Suite 5.0 software.

### Statistical evaluation

The Shapiro - Wilk test was used to check normality of the data. The obtained mean values before and after physical exercise were compared using the Student’s t-test for dependent variables. Differences at *p* < 0.05 were defined as statistically significant. Pearson correlations were calculated for all obtained results and regarded as statistically significant when *r* > 0.5 and *p* < 0.05. The Statistica 9th software (Statsoft, USA) and Excel 2007 were used.

## Results

Exercise tests carried out in all subjects demonstrated significant differences in plasma epinephrine concentrations and the expressions of the investigated genes at maximal exercise intensity (Te) compared to pre-exercise levels (Ts). Significant differences in gene expressions were also observed between Te and after 15-minute recovery (Tr). The baseline (resting) plasma E concentration of 0.40 ± 0.22 nmol·L^−1^ increased significantly after exercise to the level of 1.34 ± 0.93 nmol·L^−1^ (p <0.01). In Tr, the mean value of this hormone decreased and was not significantly different from its baseline values. No significant decrease was observed between the Ts (3.61 ± 1.46 nmol·L^−1^) and Te (1.11 ± 0.69 nmol·L^−1^) values of norepinephrine concentrations (p > 0.05).

**Figure 1.**
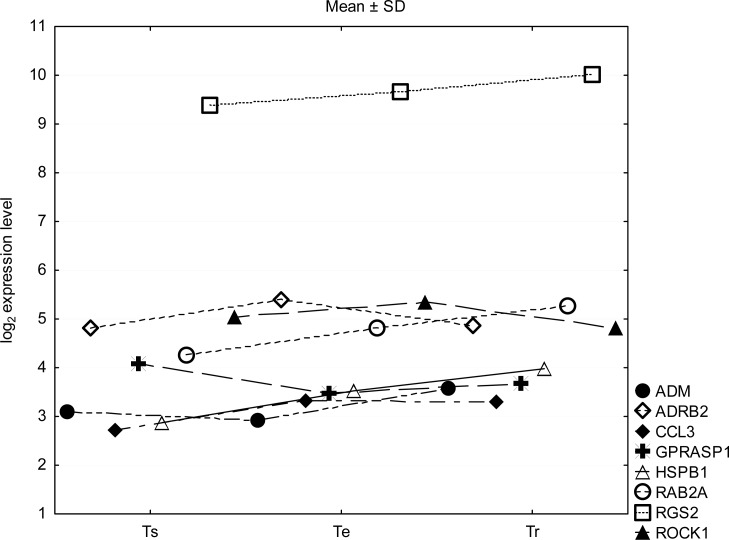
Distinguished genes of adrenergic system related in investigated road cyclist. Expression of selected genes in cyclists at rest (Ts), at maximal exercise intensity (Te), and during 15 min of recovery (Tr).

Statistical analysis of 224 mRNA (132 genes) associated with the adrenergic system determined based on the HG-U133A Affymetrix microarray analysis allowed the identification of eight genes that differentiated the particular periods of the experiment. Based on linear regression curves (Ts vs Te; Te vs Tr; Ts vs Tr) and points outside of prediction lines the following eight genes were identified: ADM (adrenomedullin), ADRB2 (β-2 adrenoceptor), CCL3 (C C motif chemokine 3), GPRASP1 (G protein receptor associated sorting protein 1), HSPB1 (heat shock protein 27 kDa), RAB2A (related to ras oncogene family), RGS2 (regulator of G-protein signalling 2) and ROCK1 (Rho combined kinase 1). The mean values and standard deviations of their expression (log_2_) in the periods of the experiment are shown in Figure 1. The highest expression was for the RGS2 gene, but its levels were not significantly different between the examination periods. It was found that only ADRB2 and RAB2A gene expression levels significantly increased in response to exercise, from 4.8 ± 0.12 and 4.27 ± 0.26 in Ts, to 5.41 ± 0.26 and 4.8 ± 0.12 in Te (p <0.05), respectively. In Tr the ADRB2 expression returned to the Ts levels, whereas the RAB2A significantly increased in comparison to baseline (p <0.02), reaching a value of 5.27 ± 0.33. Expression of ADM and HSPB1 significantly increased in Tr as compared to expression of these genes in Ts (p <0.05).

The correlations between all the investigated blood variables, hormone levels and gene expressions at different phases are presented in Table 1. Statistically significant correlations between the E concentrations and RAB2A or GPRASP1 gene expressions as well as between the expressions of RAB2A and GPRASP1 were found at rest. The post-exercise phase was characterized by positive and significant correlations between: ADRB2 and CCL3, ADM and GPRASP1, and negative between ADRB2 and RAB2A. In the recovery period and significant positive correlations were found between ADRB2 and ADM as well as between HSPB1 and ROCK1 expressions (Table 1).

## Discussion

Physical exercise is a signal to release E and NE, the main adrenergic system neurotransmitters, from the adrenal glands and synapses into the bloodstream. Changes in the level of plasma catecholamine concentration depend on, among others, the duration of exercise, the discipline of the sport, and the level of fitness (Ježova et al., 1985; Zouhal et al., 2008). In the investigated road cyclists, we observed a statistically significant increase in plasma E concentration; the values were three and a half times higher compared to the baseline. Numerous researchers have reported changes in plasma E and NE concentrations under the influence of exercise, with differences depending on training status and the exercise’s intensity (Zouhal et al., 2008; Jost et al., 1989). We did not observe statistically significant differences between plasma NE concentrations during exercise and those at rest or recovery. These results may indicate that circulating catecholamines elevation (E and NE) is more likely to be associated with metabolic adaptation than exercise-related stress (Urhausen et al., 1995 Zouhal et al., 2008). This type of response seems to be typical of competitive athletes with high VO_2max_ as were those who participated in our study. Endurance training also decreases the basal plasma NE concentration and NE : E ratio, which is associated with the reduced activity and lower β receptor density and higher α-2 receptor sensitivity of trained athletes (Jost et al., 1989). Thus, the differences in sympathoadrenergic system activation and adrenergic receptor gene expression might be of key importance for exercise adaptation in competitive athletes (Dimsdale and Moss, 1980; Zouhal et al., 2008; Fragala et al., 2011).

Our results demonstrate an exercise-induced increase of the heart rate and cardiovascular adaptation to exercise probably resulting from circulating epinephrine stimulation of the β2 adrenergic receptors in investigated athletes. These findings confirm previous observations that changes in adrenergic receptor gene expression, mainly ADRB2, play an essential role in adaptations to exercise. In the present study, we analyzed 224 mRNA associated with the adrenergic system and we found significant changes in the expression of 8 genes of the adrenergic system in response to exercise. Expressions of these genes were different at maximal exercise intensity and during 15 minutes of recovery.

The results show a significant RGS2 gene expression at rest (Ts), at maximal exercise intensity and during 15 minutes of recovery (Figure 1). However, no relationships were found between the expression of this gene and the determined catecholamine concentrations. The RGS2 protein belongs to a family of G-proteins acting as a GTPase associated with heterotrimeric Gα subunit (Vinayagam, 2011). The hemodynamic studies indicate that the RGS2 gene is involved in blood pressure regulation by acting on the central nervous system (Welch, 2010). In hypertensive patients, a reduction in RGS2 mRNA and protein level was observed compared with normotensive subjects (Semplicini et al., 2006), but hypotensive patients (Bartter’s/Gitelman’s syndrome) showed an increase of the RGS2 expression (Calo et al., 2004). RGS2 takes part in the regulation of normal vascular tone and blood pressure, as observed in knockout mice; reduction or abnormal function of RGS2 contributes to hypertension (Heximer et al., 2003).

Our results demonstrated that maximal exercise significantly increased ADRB2 and RAB2A expressions compared to resting levels. However, a significant decrease of the ADRB2 expression was observed 15 minutes after exercise. These findings are in line with the studies evidencing an increase in ADRB2 density with an exercise duration but reduction below baseline levels after exercise (Frey et al., 1985). As documented, ADRB2 is important in cardiovascular regulation at rest and during exercise. Phosphorylation of L-type Ca2+ channels via ADRB2 stimulation enhances ventricular contraction and diastolic relaxation. This mechanism could probably alter cardiovascular structure and function in response to endurance training. An increasing RAB2A gene expression may be associated with signal transduction through ADRB2 and/or with the activity of RAB2A as a GTPase. Because ADRB2 is connected with E, thus activity of RAB2A may also depend on neurotransmitter-receptor binding. The expression of the RAB2A gene in Ts was correlated with baseline plasma E concentrations (Table 1). RAB2A regulates the transport from the endoplasmic reticulum and Golgi apparatus to the cell surface via the ADRB2 (Wang and Wu, 2012), which might indicate some effect of E concentration on this process. In our study, the effect of E and its receptor was confirmed by a statistically significant correlation between E and RAB2A as well as ADRB2 and RAB2A. However, these correlations do not exclude the participation of RAB2A in the regulation of reverse transport. In our study, a relationship was found between E and GPRASP1 (GASP-1) or RAB2A gene expressions in Ts, as well as between RAB2A and GPRASP1. The GPRASP1 gene encodes for a protein belonging to the family of G protein-coupling receptors, which may modulate lysosomal sorting and functional down-regulation of variety of G-protein coupled receptors. It has been shown that strength training causes the reduction of myostatin gene expression in blood and affects the increased expression of GASP-1 (Laurentino et al., 2012).

We observed a significant negative correlation between the ADRB2 and RAB2A expression, as well as positive correlation between ADRB2 and CCL3 at maximal exercise intensity (Table 1). These findings may suggest changes in the RAB2A and CCL3 expression, which are closely associated with β2 receptors stimulation. The ADRB2 and RAB2A expression during exercise has a beneficial effect on the cardiovascular response to exercise (Fragala et al., 2011). Genes involved in inflammatory processes such as CCL3,GPRASP1, and HSPB1 were significantly stimulated after a maximal exercise test (Tr). Our study participants exhibited a positive correlation between ADM and GPRASP1 at maximal exercise intensity. Our results may suggest that exercise-induced skeletal muscle contraction, damage of muscle cell membranes, and increased inflammatory processes all affect CCL3 expression. Significant positive correlation between ADRB2 and ADM expressions and between ROCK1 and HSPB1 during a post-exercise recovery process seems to confirm the protective effect of the adrenergic system on the cardiovascular function (Yahiaoui et al., 2008). The ADM gene encodes a vasoregulatory protein which controls vascular tone and enhances myocardial contractility (Seissler, 2012). The tendency of a lower ADM expression during exercise and higher during 15 minutes of recovery, respectively might reflect low vascular resistance during endurance training.

Expression of the HSPB1 gene significantly increased during recovery (Tr). This main function of HSPB1 is to provide cytoprotection and support cell survival under stress conditions (Edwards et al., 2012). It has been observed that HSPB1 increases in healthy individuals (Tavallaie et al., 2012). This gene (HSPB1) encodes for a small protein involved in the regulation of many intracellular processes. Its functions and relationships in the cell and importance in physiology are well-established (Mymrikov et al., 2011). The presence of this gene and protein in circulation occurs in response to intense and exhaustive physical effort (Périard et al., 2012).

In Tr, we revealed positive and significant correlations between ADRB2 and ADM as well as ROCK1 and HSPB1 expressions (Table 1). The altered ROCK1 expression was observed in the cardiovascular system and in muscles. It plays an important role in the regulation of cytokine, adhesion and aggregation of platelets and lymphocytes by converting an inactive form of GDP to the active GTP (Loirand et al., 2013). Among the estimated neurotransmitters (E and NE) in the leukocytes of trained road cyclists, we stated that only E had an effect on the transcriptional activity of adrenergic system genes in Ts, which probably stimulates transcription of RAB2A and GPRASP1. Exercise activated a β2-adrenergic receptor gene, which negatively correlated with RAB2A and positively with CCL3. Furthermore, it activated a gene encoding an adrenomedullin (ADM) which also correlated with GPRASP1 in Te. In Tr, the ADRB2 was still active and correlated with ADM, as well as ROCK1 with HSPB1. Increased secretion of catecholamine initiates the “fight-or-flight” response, in which the obtained molecules are involved, but in our results, we only observed the “fight” hormone. All of these genes are associated with the response of body muscles, blood pressure, heart function, the biochemistry of G protein-coupled receptors and their effect, as well as numerous signalling pathways associated with them. It is possible that the changed expression of these genes is not just a compensation for elevated blood pressure and prevention of heart failure, as Krzeminski et al. (2006) stated. In addition, we can suggest that the determined genes play an important role in the mechanism of adrenergic response on beneficial changes in cardiovascular function due to maximum exercise in competitive cyclists.

## Conclusion

Differences in genes expression during maximal intensity exercise and a post-exercise recovery period indicate that the adrenergic response to physical effort varies depending on the investigation stage. It seems that the stimulation of immune and resistance processes is more characteristic of maximal intensity exercise phases while the postexercise recovery period is associated with the expression of those genes that exert protective effects on the cardiovascular system.

## Figures and Tables

**Table 1:** Relationships between epinephrine concentration and selected genes at rest (Ts), at maximal exercise intensity (Te), and during 15 min of recovery (Tr).

Time	Components		Regression equation	Correlation coefficient, r	*p* - value
	x	y			
Ts	E	GPRASP1	y = 2.91 + 2.97 x	0.9999	0.011
		RAB2A	y = 3.69 + 1.46 x	0.9999	0.003

	GPRASP1	RAB2A	y = 2.26 + 0.49 x	0.9999	0.007

Te	ADRB2	RAB2A	y = 7.36 – 0.47 x	− 0.9983	0.037
		CCL3	y =− 11.66 + 2.77 x	0.9992	0.025

	ADM	GPRASP1	y = 2.18 + 0.45 x	0.9999	0.007

Tr	ADRB2	ADM	y =− 0.33 + 0.80 x	0.9983	0.038
	ROCK1	HSPB1	y =− 1.96 + 1.23 x	0.9989	0.029
